# Iodoform in Dental Surgery

**Published:** 1884-04

**Authors:** C. F. W. Bodecker

**Affiliations:** New York


### IODOFORM IN DENTAL SURGERY.

BY C. F. W. BODECKER, D. D. S., M. D. S., NEW YORK.

(Continued from page 141.)

M. Schede (Centrbl. f. chirurgie, No. 3, 1882), who is known to have observed the greatest number of cases of poisoning by iodoform, describes six different forms of the same. The most common phenomena are: a considerable elevation of the temperature, up to one hundred and four degrees Fahrenheit, without noticeable disturbances of the general health of the patient, manifesting itself soon after the iodoform has been applied. The symptoms of another form, as described by M. Schede, are: great mental depression, headache, loss of appetite, and the taste of iodoform in everything taken into the mouth; the pulse is accelerated and small, but after the removal of the iodoform these symptoms soon disappear. A third variety is mentioned in which was observed a great frequency of the pulse (from one hundred and fifty to one hundred and eighty per minute), with relatively little disturbance of the patient's general health. But in the fourth form he observed in connection with frequent pulse a corresponding rise in the temperature. This form, says M. Schede, might be mistaken for septicæmia, although in iodoform poisoning the wound is perfectly aseptic, the tongue red and moist, and sensorium clear. A fifth form is mentioned, but as this occurred after extensive surgical operations, its etiology is doubtful. The symptoms of the sixth variety are: disturbances of the functions of the brain, appearing either as phenomena of acute meningitis or actual brain diseases. The symptoms of acute meningitis were met with mainly in young patients; they present themselves as an accelerated pulse, vomiting, depression of the sensorium, sometimes to a degree of complete coma, and contractions of certain groups of muscles. Schede regards it unsafe to completely fill the cavities of large fresh wounds with iodoform, as it adheres to the tissues in such a manner that, if alarming symptoms should present themselves, its removal may be found difficult.

Hoeftman (Centrbl. f. chirurgie, No. 7, 1882) is of the opinion that the danger in the use of iodoform is connected, to a certain extent, with the form in which it is applied, as in the clinic of Konigsberg, among one thousand patients treated with iodoform in the form of crystals, not a single case of poisoning was observed, although in one of them Gm. 50,0, = ʒ12, grs. 30, was used. But in two other cases, in which the iodoform had been applied in the form of a powder, death ensued, with the symptoms of mania, retention of the urine and very high pulse. In one of these cases, after the extirpation of a cancerous breast, Gm. 20,0, = ʒ5 of the iodoform powder was applied; another after ovariotomy where Gm. 25,0, = ʒ6, grs. 15, had been made use of.

Dittel (Anzeiger d. k. k. Gesellsch., d. Aerzte in Wien, No. 25, 1882), who on an average treated nearly two thousand patients annually, never observed grave symptoms of iodoform poisoning, and only in exceptional cases loss of appetite and restlessness was noticed.

Mosting (Anzeiger d. k. k. Gesellsch., d. Aerzte in Wien, No. 6, 1882) observed, in the treatment of 5,000 patients, not a single case of poisoning by iodoform. He, however, used the precaution not to fill large wounds or cavities, but merely applied a thin layer.

H. Singer (Wien Med. Press, Nos. 15, 16, 17, 18 and 19, 1882) mentions that some patients can bear enormous doses of iodoform without the slightest sign of poisoning. Although in some instances he completely filled large cavities, and mentions a case where, in a compound fracture of the femur of a boy twelve years old, at least Gm. 150,0=about ℥ivss. of iodoform had been used without evil results.

Hofmokl (Anzeiger d. k. k. d. Aerzte in Wien, No. 6, 1882), who used iodoform in 200 cases, of which fifty-six were children, observed two or three cases of poisoning.

Czerny (Wien Med. Wochenschr No. 6, 1882) regards iodoform more dangerous than carbolic acid as a dressing for wounds. He observed fatal results after the use of Gm. 40,0=about ʒ10, and very dangerous symptoms when only Gm. 6,0=90 grs. had been applied. He is of opinion that the danger of poisoning depends more upon the extent of the wound and the presence of fat than upon the quantity of iodoform employed.

Konig (Centrbl. f. chirurgie Nos. 7 and 8, 1882), who has furnished interesting statistics, states that grave consequences as a rule are not observed unless more than Gm. 10,0=about ʒiii of iodoform has been applied. Although one case is reported where only Gm. 1,0=about 15 grs. were used, which was followed by bad symptoms. The age of the patient according to Konig is of the greatest importance.

The older the patient the greater is the disposition to iodoform poisoning. In fifteen slight and thirteen severe cases of iodoform poisoning, eleven of the patients were under thirty-five years of age; six between the ages of thirty-five and fifty; four between fifty and sixty; and eleven patients were over sixty years of age. As to the prophylaxis, it is important to note carefully the frequency of the pulse immediately after the application of the iodoform, a steady high pulse is always observed prior to the development of brain symptoms, and in these instances the iodoform should be immediately removed.

In dental practice iodoform is as yet not in general use, although those practitioners who have employed it praise it very highly. As a remedy in chronic pulpitis, a capping for exposed pulps, a dressing in oral surgery, and in some instances a preventive against an acute alveolar abscess, we possess no drug which, in its action, is as certain as iodoform. Every dental practitioner knows how annoying it is to see patients with an acute alveolar abscess, especially when this occurs in teeth, the pulps of which have been dead for some time, and which, previous to the opening of the pulp chamber, had given no trouble. I know of no remedy which will prevent this as surely as the saturated solution of iodoform in ether, when used in the proper way. In some instances we can open the chamber of a pulpless tooth, which usually contains a great deal of septic matter, clean it out, fill it at once, and no trouble whatever will arise. In these cases the end of the root is encysted, and any kind of filling material, or even no filling at all, will answer the purpose. In other instances, however, when the pulp canal in the end of the root is open, and no encystment present around the apex, an acute alveolar abscess in the majority of instances follows the opening of the pulp chamber, even if no attempt has been made to enter the pulp canal with an instrument. The formation of an alveolar abscess in these instances, I believe, is due to the entrance of air into the pulp canal. I have for nearly three years been very successful in such cases, and a number of my professional friends who have pursued the same line of treatment have met with similar results. My proceeding is as follows :

I drill a hole into the tooth or filling toward the pulp chamber, until it very nearly reaches it. I fill this drill hole with a saturate solution of iodoform in ether (about ʒi of iodoform to ℥i of sulphuric ether), and very quickly, before the ether is evaporated, pierce the remaining septum of the pulp chamber. I then fill the pulp chamber loosely with a piece of cotton saturated with the iodoform solution, and temporarily seal it. This plug I allow to remain from three to five days before I attempt to clean out either the pulp chamber or the root canal. After this time has elapsed, I remove the temporary plug, together with the cotton, make the pulp canal accessible, and as straight as possible, without interfering with the strength of the tooth. I then clean out the pulp chamber, at the same time cutting away all superfluous dentine, and thoroughly rinse it out with water. I apply the rubber dam, dry the cavity, and if the canals are accessible I at once proceed to clean and fill them, in the manner to be mentioned hereafter. If, however, the tooth presents any inaccessible narrow or curved canals, such as we meet with in the buccal roots of upper molars and first bicuspids, the mesial roots of lower molars, and most of the roots of wisdom teeth, I introduce one or two drops of an aqueous solution of chloride of zinc (about forty grains to the ounce of water), and temporarily seal the cavity with a mixture of gutta percha and wax for about twenty-four hours. When I see the patient again, before I remove the temporary plug, in order to exclude the entrance of the saliva into the canals, I apply the rubber dam. Then I remove the temporary filling, apply a few drops of absolute alcohol, dry the cavity out again, and moisten it with the solution of iodoform in ether. Now I begin to clean out the pulp canals, either with Donaldson's nerve extractors, a smooth nerve broach, the temper of which has been previously drawn, a Gate's drill, or any other suitable instrument. When the canals are as clean as I can get them with instruments, I again wash them out with absolute alcohol and dry them by means of a non-barbed pivot broach, around which I wind a few fibers of cotton, which I repeat until the cotton comes out of the canal perfectly dry and clean. I then again apply a drop or two of the saturated solution of iodoform in ether, and quickly pump it into the canal. The next step is the introduction of the filling into the root canals, for which, in my opinion, there is probably no better method nor material than that mentioned by Dr. H. J. McKellop and Dr. W. C. Barrett—(Transact. Am. Dent. Ass., 1879.)

Whenever we hear of anything new, however good and practical it may appear, we adopt it in our practice with some hesitation, or even suspicion. This was the case with me before I began to fill root canals with a solution of gutta percha in chloroform. However favorable this material appeared to me then, I could not make up my mind to adopt it without first experimenting with it out of the mouth. I took two lower bicuspid roots which had just been extracted; I removed everything out of the canals by means of a burr, after which I filled one of these roots with solution of gutta percha without any further delay. The canal of the other root, however, after it was drilled out, I washed out thoroughly with absolute alcohol before the gutta percha was introduced. After two or three days, when the filling material had hardened, I split both these roots, and by placing them under the microscope found that where I had used absolute alcohol for the dehydration of the pulp canal previous to the introduction of the filling material, the dentinal canaliculi were filled for a little distance with gutta percha, whereas in the other root I could see no gutta percha in the dentinal canaliculi. The results induced me to lay aside all other filling materials for filling root canals. The method of introducing the filling is as follows: To an ounce of a rather thin solution of gutta percha in chloroform, I add about ʒi of powdered iodoform; of this solution I introduce one or two drops into the pulp canal, and with a smooth broach force it up to the apex. This solution is succeeded by very thin pieces of previously warmed gutta percha, which, by means of a little thicker instrument, are forced into the pulp canal until it is completely filled. If the foramen in the end of the root is somewhat large, I saturate a piece of cotton, wound around a smooth nerve broach, with the solution of iodoform; let the ether evaporate; dip it into the solution of gutta percha, and force it into the pulp canal up to the apex of the root, and follow this by small thin pieces of warmed gutta percha.

If the opening in the end of the root is as large as the canal, I make a shoulder as near to the apex as possible, by enlarging the pulp canal, which, in a straight, accessible root, can be done safely as follows: The exact length of the tooth I obtain with a thin Donaldson's nerve bristle, on the end of which is a very fine hook; around this instrument I wind a few fibers of cotton, about as far from the hook end as I expect the tooth to be long; I pass this instrument into and through the pulp canal, and let the little hook take hold upon the apex of the root. I then adjust the cotton in exact length with the cutting edge of the tooth, withdraw this instrument, and mark the length of the tooth upon a thin bud-shaped or round burr, with which I enlarge the pulp canal up to about one sixty-fourth of an inch from the end of the root. I then fill the root in the same manner as before described.

I have exclusively employed the above described method of filling and treating pulp canals since July, 1881, and in order to obtain an idea of its comparative value, I have classified the teeth so filled in their regular order, namely:

| UPPER | LEFT | RIGHT | LEFT | RIGHT | LOWER |
|---|---|---|---|---|---|
| Centrals | 5 | 8 | 3 | 1 | Centrals |
| Laterals | 7 | 7 | 2 | 1 | Laterals |
| Canines | 6 | 9 | 1 | 2 | Canines |
| Bicuspids I | 20 | 9 | 3 | 5 | Bicuspids I |
| Bicuspids II | 13 | 16 | 6 | 12 | Bicuspids II |
| Molars I | 14 | 9 | 15 | 12 | Molars I |
| Molars II | 9 | 9 | 12 | 7 | Molars II |
| Molars III | 3 | 2 | 2 | 1 | Molars III |
| | 77 | 69 | 44 | 41 | |

In all there are two hundred and thirty-one pulpless teeth, of which seventy-seven were in one of the stages of an alveolar abscess before the treatment was commenced. Of all of the cases treated, sixteen were followed by an alveolar abscess after the introduction of the filling material, of which seven yielded to treatment, but nine teeth had to be extracted. A short history of these is as follows:

Miss R., age about twenty-eight, good constitution. Right lower first molar, with chronic alveolar abscess; the tooth had been previously filled. The pulp canal of the anterior root was found to be inaccessible; the tooth was treated and filled in the manner described, but the abscess did not abate. After five months the tooth was extracted, when I found adhering to the anterior root a rather large pyogenic sack, but the posterior root was comparatively healthy.

Mrs. S., age about twenty-six, good constitution, left lower first molar. The tooth had been filled four years previously, and ever since presented signs of slight pericementitis. The pulp canals were found to be filled with amalgam, which was easily removed out of the posterior root, but the anterior root could not be explored. The tooth was treated and filled as described above, but without much improvement; when four months later it was removed, I found an abscess on the anterior root.

Miss K., age about twenty, very anæmic, right lower first molar, with an acute alveolar abscess; the tooth had not been filled before, but was much discolored; it was treated and filled as mentioned above, when the abscess healed up, but soon an acute abscess again developed. When the tooth was extracted, both roots were found to be corroded at their apices.

Mrs. S., age about twenty-two, good constitution, in the fifth month of pregnancy. Right lower second molar with an acute alveolar abscess. The tooth contained a large gold filling, and the pulp had died. Treatment and filling as before mentioned, but an acute abscess again developed one day after the filling; when the tooth was extracted, the roots were found to be comparatively healthy.

Mr. H., age seventy-four, very good constitution. Left lower second bicuspid with a chronic alveolar abscess. The tooth was perfectly sound, except that it was worn down upon the grinding surface; the pulp chamber was found to be very nearly obliterated by secondary dentine. The canal was treated and filled as mentioned before, but without success; when the tooth was extracted the root was found to be corroded at the apex.

Miss K., aged about seventeen, very anæmic. Left lower second molar contained a medium-sized gold filling in the grinding surface. The tooth was affected with a chronic alveolar abscess; it contained only one pulp canal, which was funnel shaped toward the apex of the root and very large.

Treatment as before, except that in the root canal a shoulder was made in the manner heretofore described, and a piece of cotton, saturated with the solution of gutta percha, was packed against the shoulder before the solid gutta percha was introduced. After three weeks the tooth had to be extracted, when it showed several large corroded concavities on the root.

Mrs. A., age thirty-five, good constitution, in the fourth month of pregnancy. The patient complained of pain in all the teeth, but particularly the right lower second molar, which was pulpless, and had been filled previously. The filling was removed, and the canals treated as above mentioned, but without success. The tooth was extracted, which, however, only afforded temporary relief; the roots were found to be comparatively healthy.

Mr. H., age forty-five, good constitution; right lower first molar contained a large oxychloride filling in its distal surface; the pulp had died, but no pericementitis was observable. The pulp chamber was small, the anterior canal quite inaccessible, and the posterior only partially so; treatment as before described. One year later a chronic alveolar abscess developed, when the filling was removed and another attempt made to open the pulp canals, but with no more success than in the first instance. The tooth was then treated with a solution of chloride of zinc for two weeks, and again filled, and the abscess treated locally, which soon healed up nicely. But after two months and a half the patient came back with the same trouble, when he insisted upon its extraction. Both roots were very flat, much curved, and the pulp canals somewhat accessible from their apices.

Mr.K., age about thirty-five, nervous constitution; right upper third molar in which the pulp had been devitalized two years previous by arsenious acid; it was temporarily filled with gutta percha, and the pulp chamber contained a piece of cotton. The tooth showed signs of a beginning pericementitis; neither of the canals was accessible, nor could be made so on account of the position of the tooth. It was treated and filled as mentioned above, but about one month later an acute alveolar abscess developed, when it had to be extracted.

To sum up the results, we come to the conclusion that teeth of which the alveolus and surrounding pericementum have not been destroyed too far by chronic inflammation, and which contain accessible pulp canals, can as a rule be saved by the method mentioned above. As exceptions to this may be mentioned systemic disorders, which bring about disturbances in vascular currents, viz., pregnancy, pyorrhœa alveolaris, etc. I have observed that pulpless teeth, when affected with the latter disease, in the majority of instances will be followed by an acute alveolar abscess after the pulp canal has been filled, unless an encystment of the apex of the root is present.

This shows that iodoform is a valuable remedy in the treatment of pulpless teeth. It is, however, not less so in the treatment and capping of exposed pulps, but as this subject is very important and cannot be summarily treated, I prefer to write upon this subject at some future time, and for the present merely allude to it, as in a former paper. (Dental Cosmos, 1882.)

Since July, 1881, I have capped 133 exposed pulps, of which ninety-seven were capped with iodoform, and thirty-six with a five or ten per cent solution of carbolic acid mixed with oxide of zinc, succeeded by a layer of Witzel's carbolic acid cement,* over which I

*The formula for Witzel's carbolic acid cement is as follows:

℞ Acid. carbol., 5·0;
Alcohol. absol., 2·0;
Aq. dest., 40·0;
Glycerinæ, 20·0.

Mix, and add an equal volume of the zinc chloride used in preparing oxychloride fillings. To a sufficient quantity of this mixture add oxide of zinc to give it the necessary consistence for filling.

generally place oxyphosphate or gutta percha. As far as I am able to state I have met with thirteen failures out of the 133 pulps capped. Of these were three teeth in the mouths of pregnant females, four in wisdom teeth, one in the distal surface of a second molar, two in the distal, and one in the mesial surfaces of first molars, and two in distal surfaces of bicuspids.

The iodoform in shape of a powder is not well adapted for capping pulps; I have therefore used it in three different combinations:

I. In the form of a paste (Paschkis).

℞ Iodoform pulv.,
Kaolin pulv., aa 4·00,
Acid. carbol. cryst., 0·50.

Mix; add sufficient glycerine to form a paste; then add ol. menth. pip., gtt. x.

II. Iodoform powder mixed with oil of eucalyptus; and—

III. Iodoform powder rubbed up with vasseline.

